# Acute appendicitis in children: is preoperative hyponatremia a predictive factor of perforation/gangrene? A prospective study

**DOI:** 10.1007/s00383-023-05561-4

**Published:** 2023-10-10

**Authors:** Ahmed Elgendy, Mohammad Gharieb Khirallah, Mohamed Elsawaf, Hussam S. Hassan, Mohamed Ghazaly

**Affiliations:** 1 Surgical Oncology Unit, Department of Surgery, Faculty of Medicine, Tanta University, 35 Ali Beek Elkbeer Street, Tanta, 31515 Egypt; 2 Pediatric Surgery Unit, Department of Surgery, Faculty of Medicine, Tanta University, Tanta, Egypt; 3 Department of Surgery, Faculty of Medicine, Jouf University, Aljouf, Saudi Arabia

**Keywords:** Acute appendicitis, Children, Hyponatremia, Perforation

## Abstract

**Purpose:**

Distinguishing between perforated/gangrenous from uncomplicated appendicitis in children helps management. We evaluated hyponatremia as a new diagnostic marker for perforated/gangrenous appendicitis in children.

**Methods:**

A prospective study including all children with acute appendicitis who underwent appendectomy at our institution from May 2021 to May 2023. Medical history and clinical criteria were analyzed. All blood samples were taken upon admission including serum inflammatory markers and electrolytes. Patients were divided into two groups (Group I: uncomplicated and Group II: perforated/gangrenous), and data between both groups were compared.

**Results:**

The study included 153 patients [Group I: 111 (73%), Group II: 42 (27%)]. Mean serum sodium concentration in children with perforated/gangrenous appendicitis was significantly lower compared to children with uncomplicated appendicitis (131.8 mmol/L vs. 138.7 mmol/L; *p* < 0.001). The ROC curve of preoperative sodium level to differentiate between perforated/gangrenous and uncomplicated appendicitis revealed an AUC of 0.981. The cut-off-value of sodium level < 135 mmol/L identified perforated/gangrenous appendicitis with a sensitivity of 94% and a specificity of 91% (*p* < 0.001). Predictive factors of perforated/gangrenous appendicitis were: age less than 5 years (12% vs. 3%; *p* = 0.02), experiencing symptoms for more than 24 h (100% vs. 58%; *p* < 0.001), body temperature more than 38.5 °C (52% vs. 13%; *p* < 0.001), a serum sodium level less than 135 mmol/L (90% vs. 6%; *p* < 0.001), and a CRP serum level more than 50 mg/L (71% vs. 17%; *p* < 0.001).

**Conclusions:**

Hyponatremia, upon admission, is a novel, objective biochemical marker that can identify perforated/gangrenous appendicitis in children. We advocate that the assessment of serum sodium level should be added to the diagnostic algorithm in children with suspected acute appendicitis. Surgical intervention in patients with hyponatremia should not be delayed, and non-operative management should be avoided.

## Introduction

Acute appendicitis is a common indication for emergency abdominal surgery in children [1]. Its severity ranges from mild inflammation to gangrene with perforation, and localized or generalized intra-abdominal peritonitis [2]. Patients with perforated appendicitis have an increased risk of post-operative morbidities such as paralytic ileus, abscess formation, and even death in rare cases [3]. A longer period of symptoms and a significantly higher incidence of perforation is reported in children, with a perforation percentage of 31.8–45.8% [4, 5]. Consequently, early diagnosis is crucial in children, however, it remains a challenge as some symptoms are atypical in younger children [6].

An interdisciplinary diagnostic approach (including clinical examination, imaging scans, scoring systems, and laboratory tests) can slightly reduce the perforated appendicitis rates in children [7]. Previous studies have identified certain factors that could be indicative of complicated appendicitis, including age younger than 5 years, experiencing symptoms for more than 24 h, having a white blood cell count (WBC) greater than 12 × 10^9^, and a C-reactive protein (CRP) level greater than 10 mg/L [8–10]. Despite such diagnostic advances, the initial misdiagnosis is about 28–57% in children below 12 years of age [1, 8]. This leads to a delay in the detection, and novel objective laboratory markers are still required to facilitate precise and swift diagnosis.

Hyponatremia upon admission has been linked to gangrenous cholecystitis, gangrenous soft tissue infections, and colonic perforation. The pathophysiology between hyponatremia and the aforementioned infections is still unclear, but it is mostly mediated by vasopressin and Interleukin 6 [8, 9]. Recent studies suggest that hyponatremia may serve as a marker for the differentiation between perforated and non-perforated acute appendicitis [11–13]. Therefore, our study aims to explore the potential of hyponatremia as a new biochemical marker for children with perforated/gangrenous appendicitis, which could impact the decision to proceed with an urgent surgery.

## Patients and methods

### Study design

This was a prospective study that included all children with acute appendicitis who underwent appendectomy at our university institution and its affiliated hospitals between May 2021 and May 2023. Patients older than 18 years or with chronic endocrine diseases, or who had a negative appendectomy, or who were treated by abscess drainage were excluded from the enrollment. Patients’ guardians signed informed consent at the time of admission as per our center guidelines. The study proposal was reviewed and approved by the research ethics committee board of our institution (IRB0010038—approval number: 34794/7/21).

Meticulous medical history (symptoms and duration) was obtained from children and/or their parents. A careful clinical examination was performed to document all clinical data. Abdominal ultrasound was conducted for all patients as the imaging modality of choice. In some children with equivocal presentations, computed tomography (CT) scan was performed to confirm the diagnosis. The timing of blood sampling for laboratory tests was uniform in all patients. All samples were withdrawn at the time of admission and before the administration of any intravenous fluids. Complete blood picture, CRP, glucose level, and serum electrolytes were assessed in all children. Hyponatremia was defined as a concentration of sodium in the serum less than 135 mmol/L.

### Surgical procedure

The operative approach of appendectomy (conventional open or laparoscopic) was decided according to the preference of the operating surgeon. Conventional open technique was done through the classic McBurny or modified Lanz incisions [14]. Laparoscopic appendectomy was conducted using the classical three ports approach, a 10-mm umbilical port for the camera and two 5 mm working ports [15]. Following pneumoperitoneum and peritoneal cavity exploration, the mesoappendix was sealed by electrocautery close to the appendix wall. The appendicular base was closed using hand-made Vicryl suture loops or hem-o-lok clips [15]. The appendix was extracted via the umbilical port using a homemade glove retrieval bag. Irrigation of saline, suction, and drain insertion were performed in patients with complicated appendicitis. Operative time was estimated as the time between the first incision and the last skin suture. Intraoperative preliminary diagnosis of perforation/gangrene was based on the presence of a hole in the appendix or the presence of appendiceal wall gangrene or intraperitoneal pus; however, the final result and grouping were based on the histopathological report of the resected appendix. All specimens were sent for histopathological examination to confirm the presence or absence of appendiceal wall necrosis (gangrene), and gross or microscopic perforation. Perforation was assigned when a transmural defect in the appendiceal wall was found, and gangrene was considered when ischemic areas in the appendix and transmural necrosis were observed [16]. Suppurative, uncomplicated appendicitis was defined by the presence of transmural infiltration with granulocytes, serositis, and microabscesses without gangrene or perforation [17]. The patients were divided into two groups as per the severity of appendicitis: Group I: included children with uncomplicated appendicitis and Group II: contained children with gangrenous/perforated appendicitis.

### Post-operative care and follow-up

Antibiotic therapy was administered to all children with gangrenous/perforated appendicitis. The patients with uncomplicated appendicitis received antibiotics if intraperitoneal free fluid was observed during the surgical procedure or if they had fever attacks in the early post-operative days. Third-generation cephalosporin and metronidazole were the empirical therapy and were given as per the patient’s age and weight. Specific antibiotics were administered according to the cytological analysis of the intraperitoneal aspirate during surgery. Patients were discharged after pain control, resuming oral feeding, and in the absence of fever. Children were followed up at the outpatient clinic after 1 week of discharge, to examine the wound and assess the general condition. Another final follow-up visit was scheduled after 3 weeks.

### Outcome measures and statistical analysis

A significant correlation between hyponatremia upon admission and intraoperative confirmation of gangrenous/perforated appendicitis was the primary outcome measure. Detection of other potential predictors (such as age, gender, duration of symptoms, fever, WBC count, and CRP level) for complicated pediatric appendicitis was the secondary outcome.

Clinical, laboratory, and operative data were collated in a single sheet, and grouped as aforementioned. We performed the statistical analysis using SPSS (statistical package for social science, version 23.0). The quantitative variables were expressed as mean ± standard deviation (SD). Student’s t and Chi-square tests were used to compare the data of both groups. The authors conducted the receiver operating characteristic (ROC) curve to indicate the best cut-off-value for the prediction of complicated appendicitis and values of area under the curve (AUC), specificity, and sensitivity were estimated. The predictive factors of gangrenous/perforated appendicitis were analyzed using univariate logistic regression. Statistical significance was approved at a *p* value of less than 0.05. Statistically significant variables in the univariate analysis were enrolled into a multivariate logistic regression model.

## Results

A total of 153 patients were identified and included in the analysis. Group I consisted of 111 patients (73%) with uncomplicated appendicitis (67 males and 44 females). Group II contained 42 patients (27%) with perforated/gangrenous appendicitis (23 males and 19 females). All patients in Group II (*n* = 42) had appendiceal perforation that was confirmed by post-operative histopathological examination, and 31 patients of the same group had additional appendiceal wall necrosis besides perforation. The patients in Group II were significantly younger than those in Group I (10.8 ± 3.3 years vs. 12.3 ± 3.1 years; *p* = 0.012). There were no significant differences between the patients with perforated/gangrenous and uncomplicated appendicitis with regard to gender, nausea and vomiting, Blumberg sign (rebound tenderness), and rates of complications. Significant differences were seen between both groups concerning the duration of clinical symptoms (64.5 h vs. 22.7 h; *p* < 0.001), body temperature (38.9 °C vs. 37.4 °C; *p* < 0.001), the duration of surgical procedure (52.4 min vs. 33.7 min; *p* < 0.001), and the duration of hospital stay (6.5 days vs. 2.4 days; *p* < 0.001). The patients’ demographics, clinical criteria, and operative outcomes of both groups are summarized in Table 1.

**Table 1:** Patients’ demographics, clinical criteria, and operative outcomes of both groups

	Group I (uncomplicated appendicitis)	Group II (perforated/gangrenous appendicitis)	*p *value
Number of the patients	111	42	–
Demographics			
Age (years)	12.3 ± 3.1	10.8 ± 3.3	**0.012**
Gender (M/F) (%)	67/44 (60.4% / 33.6%)	23/19 (54.8% / 45.2%)	0.530
Clinical criteria			
Duration of symptoms (h)	22.7 ± 10.9	64.5 ± 28.3	**< 0.001**
Vomiting, *n* (%)	74 (66.7%)	33 (78.6%)	0.152
Body temperature (°C)	37.4 ± 0.63	38.9 ± 1.1	**< 0.001**
Blumberg sign, *n* (%)	103 (92.8%)	42 (100%)	0.074
Surgical procedure			
Open	17 (15.3%)	16 (38%)	
Laparoscopic	94 (84.7%)	26 (62%)	**0.002**
Mean operative time (min)	33.7 ± 11.3	52.4 ± 14.8	**< 0.001**
Intraoperative complications	–	–	–
Outcomes			
Post-operative complications, *n* (%)	6 (5.4%)	3 (7.1%)	0.684
Hospital stay (days)	2.4 ± 0.85	6.5 ± 2.1	**< 0.001**

Bold indicates the significant value in the statistical analysis

**Fig. 1:**
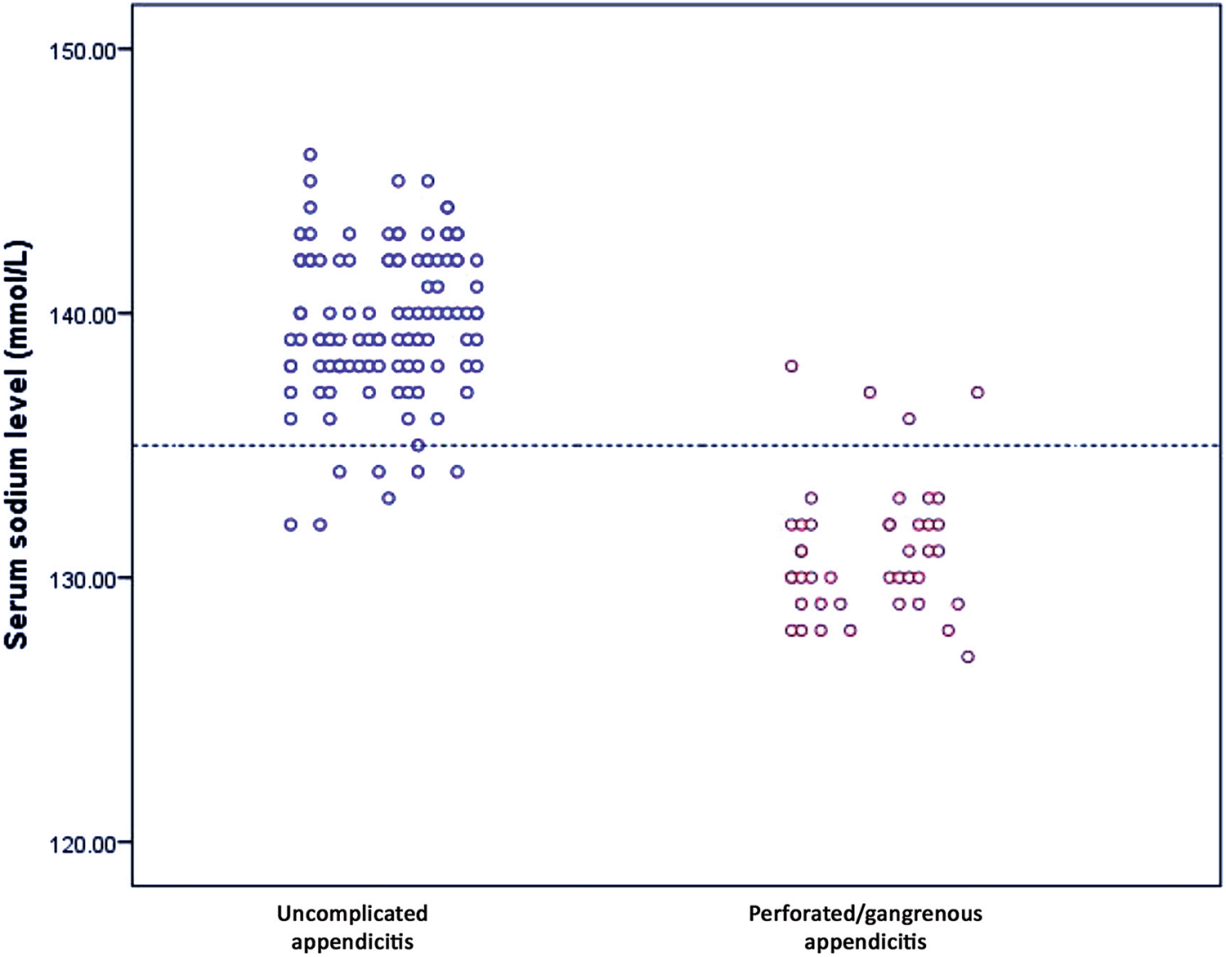
Comparison of serum sodium level upon admission between patients with uncomplicated (*n* = 111) and perforated/gangrenous (*n* = 42) appendicitis. The dotted line refers to the cut-off-value (less than 135 mmol/L)

The analysis of preoperative laboratory markers between children with perforated/gangrenous and uncomplicated appendicitis revealed a statistically significant difference with regard to WBC count (18.3 × 10^9^/L vs. 13.9 × 10^9^/L; *p* < 0.001) and CRP level (118.4 mg/L vs. 36.7 mg/L; *p* < 0.001) as shown in Table 2. The mean serum sodium level in children with perforated/gangrenous appendicitis was significantly lower when compared to the level in children with uncomplicated appendicitis (131.8 mmol/L vs. 138.7 mmol/L; *p* < 0.001) as demonstrated in Fig. 1.

**Table 2:** Preoperative laboratory data of the patients in both groups

Laboratory tests (mean ± SD)	Group I (uncomplicated appendicitis) *n* = 111	Group II (perforated/gangrenous appendicitis) *n* = 42	*p* value
WBC count (× 10^9^/L)	13.9 ± 4.6	18.3 ± 5.7	**< 0.001**
CRP level (mg/L)	36.7 ± 16.9	118.4 ± 52.3	**< 0.001**
Sodium (mmol/L)	138.7 ± 2.5	131.8 ± 2.2	**< 0.001**
Potassium (mmol/L)	4.2 ± 0.5	4.1 ± 0.6	0.298
Glucose (mmol/L)	5.8 ± 2.1	5.4 ± 0.81	0.232

Bold indicates the significant value in the statistical analysis

**Fig. 2:**
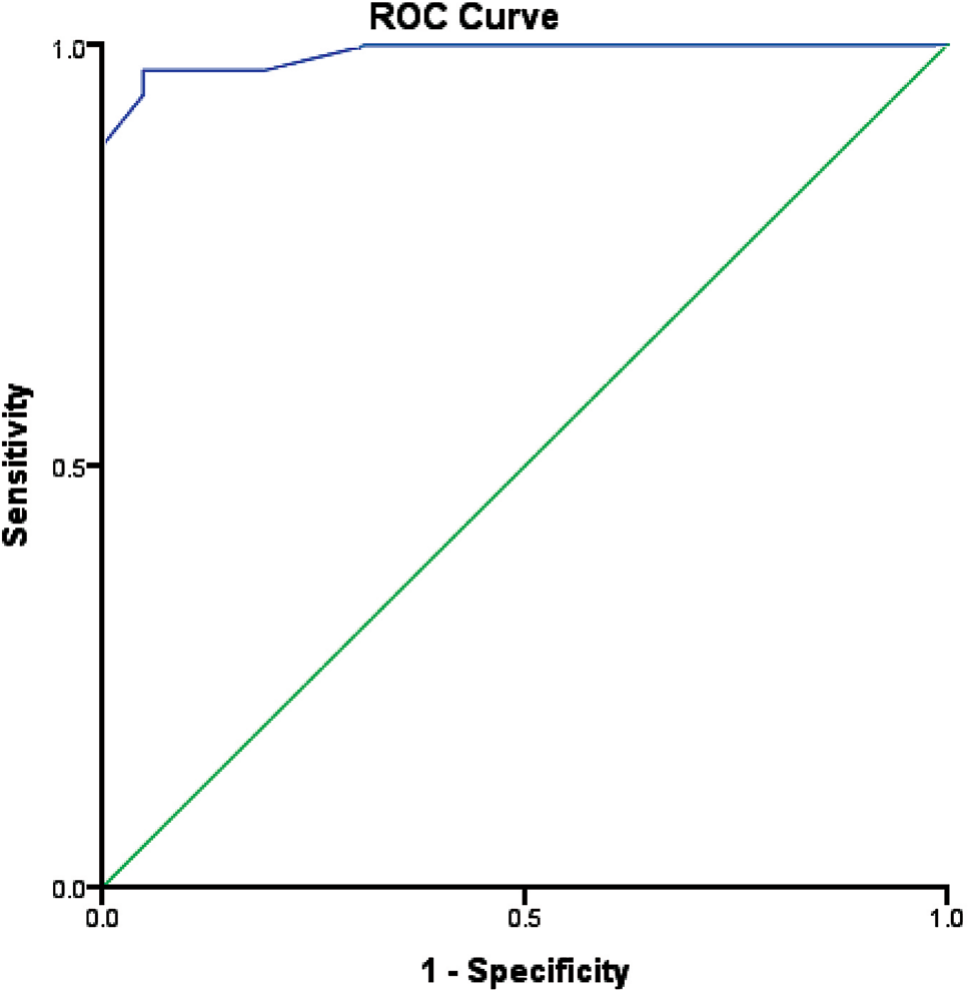
The ROC curve for serum sodium level as a predictor of perforated/gangrenous appendicitis. (AUC: 0.981; *p* < 0.001)

The ROC curve of preoperative serum sodium level for the differentiation between perforated/gangrenous and non-complicated appendicitis documented the AUC of 0.981 as shown in Fig. 2. The cut-off-value of serum sodium level of less than 135 mmol/L distinguished perforated/gangrenous appendicitis versus uncomplicated appendicitis with a sensitivity of 94%, a specificity of 91%, and an overall accuracy of 90% (*p* < 0.001).

The patients with perforated/gangrenous appendicitis were more commonly to be aged less than 5 years (12% vs. 3%; *p* = 0.02), experienced symptoms for more than 24 h (100% vs. 58%; *p* < 0.001), with body temperature more than 38.5°C (52% vs. 13%; *p* < 0.001), had a serum sodium level less than 135 mmol/L (90% vs. 6%; *p* < 0.001), and a CRP serum level more than 50 mg/L (71% vs. 17%; *p* < 0.001). There was no statistically significant difference regarding WBC count more than 12 × 10^9^/L (81% vs. 74%; *p* = 0.361). Table 3 documents the predictive factors of perforated/gangrenous appendicitis by univariate analysis. In the multivariate logistic regression model, patients with clinical symptoms for more than 24 h were over five times more common to have perforated/gangrenous appendicitis (*p* < 0.001). Patients with hyponatremia (*p* = 0.008) and patients aged less than 5 years (*p* = 0.012) were over three and two times to have perforated/gangrenous appendicitis, respectively. The results of the multivariate analysis are summarized in Table 4.

**Table 3:** Predictive factors of perforated/gangrenous appendicitis by the univariate analysis

Factor *n* (%)	Group I (uncomplicated appendicitis) *n* = 111	Group II (perforated/gangrenous appendicitis) *n* = 42	*p* value
Age (less than 5 years)	3 (2.7%)	5 (11.9%)	**0.02**
Duration of clinical symptoms more than 24 h	65 (58.5%)	42 (100%)	**< 0.001**
Hyponatremia (Na less than 135 mmol/L)	7 (6.3%)	38 (90.5%)	**< 0.001**
WBC count more than 12 × 10^9^/L	82 (74%)	34 (81%)	0.361
CRP level more than 50 mg/L	19 (17.1%)	30 (71.4%)	**< 0.001**
Body temperature more than 38.5 °C	14 (12.6%)	22 (52.4%)	**< 0.001**

Bold indicates the significant value in the statistical analysis

**Table 4:** Results of the multivariate logistic regression model

Parameter	Hazard ratio	95% CI	*p* value
Duration of clinical symptoms more than 24 h	5.2	2.9–7.3	**< 0.001**
Hyponatremia (Na less than 135 mmol/L)	3.0	1.8–4.6	**0.008**
Age less than 5 years	2.2	1.3–3.6	**0.012**
CRP level more than 50 mg/L	1.6	0.67–1.9	0.093
Body temperature more than 38.5 °C	1.3	0.85–1.4	0.162

Bold indicates the significant value in the statistical analysis

## Discussion

The current study assesses the role of hyponatremia, in pediatric patients presenting with acute appendicitis, as a new preoperative marker to distinguish between perforated/gangrenous and uncomplicated appendicitis. This subsequently could impact the decision to proceed with surgical intervention or to treat the patient using non-operative management. It is crucial for pediatric surgeons to anticipate the likelihood of gangrenous/perforated appendicitis prior to surgery, as doing so can adjust their management approach. Moreover, this provides guidance to the parents on what to expect in terms of post-operative recovery, potentially encountered complications, and the length of hospital stay. Previous studies have linked hyponatremia to increased mortality rates in adult patients with gangrenous cholecystitis or bowel ischemia due to mechanical intestinal obstruction [18, 19]. According to Swart and colleagues, this may be due to the release of antidiuretic hormone triggered by the production of cytokines (IL-1b and IL-6) during severe inflammatory reactions [20]. The pathophysiology of hyponatremia and complicated appendicitis is likely mediated by the same cytokines, as suggested by recent publications [8, 9].

In our prospective study, we observed a correlation between hyponatremia upon admission and perforation/gangrene in patients presenting with acute appendicitis. The serum sodium level in children with perforated/gangrenous appendicitis was significantly lower when compared to those with uncomplicated appendicitis. Additionally, a preoperative sodium level of less than 135 mmol/L was a predictor with high sensitivity and specificity for complicated appendicitis. We also concluded by multivariate analysis that children with hyponatremia were over three times to have perforated/gangrenous appendicitis. Lindestam and co-workers reported in their prospective study the same significant correlation, with a sensitivity of 82% and a specificity of 87% at a cut-off value of less than 136 mmol/L. They also declared that children with preoperative hyponatremia had a 15-fold increased risk of perforated appendicitis [12]. Similar results were observed in a prospective clinical trial by Pogorelić et al. [21] with a cut-off of less than 135 mmol/L that showed a sensitivity and specificity of 94.7% and 88.5%, respectively. Pham and colleagues demonstrated in their retrospective study that hyponatremia at the time of admission was 3.1 times more commonly associated with perforated appendicitis in the pediatric population [9]. However, Besli et al. [22] reported in a retrospective cohort that low plasma sodium levels had no significant correlation with perforated appendicitis in children, with a specificity of 31.1%.

We demonstrated in the present study that children with perforated/gangrenous appendicitis were more likely to be younger than 5 years of age. Such an observation was also reported by others [8, 9, 21]. Moreover, a significant association was observed in our cohort study between perforation/gangrene and experiencing symptoms for more than a day, raised level of CRP, and increased body temperature, and this correlation was documented by previous studies [6, 12]. Our study presents hyponatremia as a new indicator for perforated/gangrenous appendicitis in addition to a confirmation of the previously known predictors. The authors of this study strongly recommend that serum sodium level should be assessed at the time of admission with other routine inflammatory markers. Measuring plasma sodium concentration is not only a promising effective means of distinguishing between perforated/gangrenous and non-complicated cases of acute appendicitis, but it is also a low-cost, easily accessible test that can be performed for all children in the emergency department [12].

Currently, there is a global interest in non-operative management with antibiotic medications only for diligently selected children with acute appendicitis [23, 24]. The initial findings reported a success rate of about 90% among children with non-perforated appendicitis [25–27]. However, the conservative approach for pediatric patients with perforated appendicitis showed less successful results [9]. Therefore, the early determination of cases with perforation/gangrene is vitally important as these children should undergo a prompt surgical intervention and non-operative treatment should be avoided. Based on the aforementioned data, we believe that hyponatremia upon admission has a role in the diagnostic plan to identify perforation/gangrene and another one in the therapeutic process to decide the management strategy. We could argue that the patients with normal sodium levels should be considered for conservative management.

The relatively small number of patients and being of a single institutional experience were the main limitations of our study. Additionally, the prior use of antipyretic medications by patients’ families before presenting to the hospital might have affected the reliability of body temperature measurements, and the data of children who were treated conservatively were not included. On the other hand, the prospective aspect of this study enhances its validity in addition to the uniform timing of preoperative serum sodium measurement. Eventually, before being included in standardized protocols for detecting perforated/gangrenous appendicitis in children, our findings need to be confirmed in larger, prospective multicenter studies.

## Conclusion

Hyponatremia, upon admission, is a novel, objective biochemical marker that can identify perforated/gangrenous appendicitis in pediatric age. We advocate that the assessment of serum sodium level should be added to the diagnostic algorithm in children with suspected acute appendicitis. Surgical intervention in patients with hyponatremia should not be delayed, and non-operative management should be avoided.

## Data Availability

The dataset used and/or analyzed during this study is available from the corresponding author on a reasonable request.
